# Holistic Vision of Cardiovascular Complications in Chronic Kidney Disease

**DOI:** 10.31083/j.rcm2402057

**Published:** 2023-02-08

**Authors:** Nazareno Carullo, Michele Andreucci, Giuseppe Coppolino

**Affiliations:** ^1^Renal Unit, University “Magna Graecia” of Catanzaro, 88100 Catanzaro, Italy

Chronic kidney disease (CKD), which can result in the total loss of renal function with the need for substitutive therapy with peritoneal dialysis or haemodialysis or renal transplantation, has an ‘epidemic’ diffusion, mainly attributed to progressive aging of the general population. In the USA, CKD is the ninth leading cause of death and affects more than 10% of the adult population [1].

Low estimated glomerular filtration rate (eGFR) is a strong, independent predictor of all-cause mortality and cardiovascular (CV) diseases [2, 3], which are the first cause of morbidity and mortality in renal patients [1]. The increased CV risk in patients with CKD is related to both traditional (hypertension, diabetes mellitus, obesity, smoking, etc.) and non-traditional uremia-specific risk factors (Table 1), such as inflammation, oxidative stress, advanced glycation end-products (AGEs), vascular calcification and uremic retention solutes, whose prevalence and relevance increase with worsening renal function [4, 5, 6]. The kidney and heart are strictly linked by a bidirectional pathophysiological mechanism that may primarily result in dysfunction of one or the other. This intrinsic and complex interaction led to the definition of cardiorenal syndrome (CRS) [7]. In this context, Scagliola and Brunelli have investigated the relationship between systemic hypoperfusion and venous congestion in CRS [8]. A determinant role is played by the increased intra-abdominal pressure that is a frequent cause of urinary retention and increased cardiac filling pressures leading to worsening of heart failure (HF) [9]. Renal and cardiac damage is mediated by specific biochemical pathways. Downregulation of the nitric oxide pathway, long-terminal activation of the Renin-Angiotensin-Aldosterone (RAAS) and the sympathetic systems, produce an intense glomerular vasoconstriction with reduced GFR, vascular remodelling and proliferation, and endothelial dysfunction. Due to the pathogenetic complexity of CRS and different organ involvement, treatment is currently a challenge. Beyond the well-known cornerstones of therapy, such as diuretics, other compounds are still studied, such as recombinant human brain natriuretic peptide (Nesiritide), $V_{2}$ recepetor antagonist (Tolvapatan) and Relaxin. An increasing important role was given to ultrafiltration with numerous advantages over diuretic therapy, despite possible severe complications mainly related to the need of a central venous access. Currently, there is insufficient evidence showing its superiority compared with medical treatment [10].

**Table 1.** **Cardiovascular risk factors in chronic kidney disease (CKD) patients**.

Traditional Risk Factors	Non-traditional Uremia-Specific Risk Factors
Age	Oxidative stress
Gender	Inflammation
Smoking	Advanced Glycation End-Products
Diabetes Mellitus	Gut Dysbiosis
Hypertension	Endothelial Dysfunction
Dyslipidaemia	Anaemia
Insulin Resistance and Atherosclerosis	Secondary Hyperparathyroidism and Mineral and Bone Disorder
Obesity	Vascular Calcification

Among the various forms of CRS, type 1 may result from several clinical scenarios including cardiopulmonary bypass (CPB) surgery-associated Acute Kidney Injury (AKI), which is a frequent complication in these patients (up to 30 %), but often late recognized and poorly prevented. For these reasons, investigation of early biomarkers of acute kidney damage are of increasing interest. In a recent pilot study, Bolignano *et al*. [11] showed that selenoprotein-p1 (SEPP1), a circulating, anti-oxidant selenium transporter, could be a useful predictive biomarker of AKI in patients undergoing CPB. Specifically, circulating SEPP1 release occurs early (at 4 and 8 hours) in patients with AKI; conversely, no difference was noted 12 hours after surgery compared with the control group. After CPB, early SEPP1 measurement might have a great potential for identifying cardiac surgery patients at risk of developing in-hospital AKI.

Another distinctive population at high CV risk includes kidney transplant (Ktx) patients in which CV disease remains the leading cause of death, even more than infections and malignancies [12]. The increase in carotid intima-media thickness (IMT) is a good indicator of incipient atherosclerosis. In this setting, another possible early predictor was examined in a population of Ktx recipients [13]: Cathepsin-K (CatK), a cysteine protease with elastase and collagenase activities involved in vascular remodelling, as well as in the pathogenesis of progressive atherosclerosis [14, 15]. This study found that CatK levels in Ktx recipients were lower compared with those in patients on chronic haemodialysis (HD) and comparable to those measured in healthy controls. Therefore, Ktx seems to restore to normal the altered CatK levels that occur in HD patients. Interestingly, an inverse and clear association was noted between CatK and mean IMT values. For these reasons, CatK appears to be a promising marker for early detection of incipient atherosclerosis. CatK, together with other anamnestic, clinical, biochemical, and instrumental data could allow stratification of CV risk in this category of patients. Significantly, a close correlation between CatK levels and tele-diastolic volume of the left ventricle has been shown. It can be speculated that CatK is involved in the cardiac morpho-functional adaptation that develops in CKD patients and surely deserves further investigation.

It seems that Ktx, especially if long-standing, allows to reduce the deleterious effects induced by the uremic milieu on the cardiovascular system. Zhai G *et al*. [16], observed that coronary artery disease (CAD) in a group of patients with functional renal grafts is less severe than in long-term dialysis. The same observation was noted in Ktx patients who had a significatively longer duration of end stage kidney disease (ESRD), demonstrating a protective effect of transplantation against further development of vascular calcification.

So, it is crucial to prevent and treat CV diseases in CKD patients starting from the reduction of risk factors and, in particular, focusing on lifestyle factors. It is known that physical activity (PA) is associated with a reduced risk of CV death and mortality in CKD patients, independently from diabetes mellitus [17]. Ning/Li *et al*. [18] have demonstrated the association between a moderate leisure-time physical activity intensity (LTPA) (for longer than 30 minutes) and a reduced risk of mortality in general CKD patients. Conversely, vigorous LTPA had a noticeable effect on mortality and the same moderate LTPA showed no benefits for patients with CV complications.

CKD is a condition that affects over 10% of the general population worldwide, involving more than 800 million individuals and has emerged as one of the most prominent causes of death and suffering in the 21st century [19] with cardiovascular death being the leading cause of death. Despite the high prevalence of both CKD and CV diseases, there are significant limitations in our current knowledge about them and especially in the available weapons/armamentarium for managing this fragile population. Therefore, it is desirable that with the upcoming advent of new therapies, the high CV risk present in patients with CKD will be modified in the future.

Considering the scientific relevance of this topic, this special issue of Reviews in Cardiovascular Medicine has brought together the clinical and experimental experience of several scientists, who have focused their aims in advancing the study of new biomarkers of cardiovascular risk, new insights into clarifying underlying pathogenic mechanisms, and present and current therapeutic perspectives.
